# The Efficacy of a Super-Resolution Reconstruction Radiomics Model Based on T2WI for Predicting Placenta Accreta Spectrum Disorders: A Multicenter Study

**DOI:** 10.2174/0115734056451296260627050209

**Published:** 2026-07-09

**Authors:** Yiwei Mou, Changyi Guo, Xirong Zhang, Dong Han, Nan Yu, Xiaoqi Huang, Yiming Li

**Affiliations:** 1 Department of Medical Techniques, Shaanxi University of Chinese Medicine, Xianyang, 712000, China; 2 Department of Radiology, The Second Affiliated Hospital of Shaanxi University of Chinese Medicine, Xianyang, 712000, China; 3 Department of Radiology, The Affiliated Hospital of Shaanxi University of Chinese Medicine, Xianyang, 712000, China; 4 Department of Radiology, Yan’an University Affiliated Hospital. Yan’an, 716000, China; 5 Department of Radiology, First People’s Hospital of Shangqiu, Shangqiu, 476000, China

**Keywords:** Super-resolution reconstruction, Radiomics, Placenta accreta spectrum disorders, Magnetic resonance imaging, Deep learning, Predictive model, Multicenter study

## Abstract

**Introduction/Objective:**

Placenta accreta spectrum (PAS) disorders threaten maternal and fetal health. This multicenter study aimed to evaluate whether super-resolution reconstruction (SRR) of T2-weighted magnetic resonance images improves the diagnostic performance of a radiomics model for predicting PAS compared with conventional images.

**Methods:**

We retrospectively analyzed 603 suspected PAS cases from three centers. Center A (n=480, 224 PAS *vs*. 256 non-PAS) served as the training dataset; Centers B (n=66) and C (n=57) were external validation sets. Deep learning-based SRR generated 2× and 4× super-resolution T2WI. An automated nnUNet model segmented the placenta. From each resolution, 107 radiomics features were extracted and reduced by LASSO regression. Three classifiers (KNN, AdaBoost, and Gradient Boosting) were developed. Model performance was assessed using AUC and DeLong’s test.

**Results:**

The nnUNet segmentation achieved Dice coefficients of 0.863 and 0.883 on the two external sets. In the training set, the Gradient Boosting model on 4× images yielded the highest AUC of 0.874 (95% CI: 0.8441-0.9047). However, DeLong tests showed no statistically significant differences among the 1×, 2×, and 4× models for any classifier (all P > 0.05). External validation AUCs ranged from 0.542 to 0.724, indicating only moderate generalizability.

**Discussion:**

Although SRR significantly enhanced image resolution, it did not provide incremental diagnostic value for PAS prediction within the radiomics framework. The diagnostic information relevant to PAS may be adequately captured at original resolution, or the radiomics features used are insensitive to SRR-enhanced textural changes.

**Conclusion:**

Radiomics models based on 2× or 4× super-resolution T2WI were not superior to those based on pre-super-resolution images for predicting PAS. Routine SRR is not recommended for this clinical application.

## 1 INTRODUCTION

Placenta accreta spectrum (PAS) disorders are a group of conditions characterized by abnormal placental adherence, encompassing placenta accreta, increta, and percreta [1-3]. These disorders pose a significant threat to maternal and infant health. Previous research indicates that radiomics can potentially improve patient management and clinical decision-making by revealing disease characteristics not visible to the human eye [4, 5]. In recent years, the number of radiomics studies aimed at predicting PAS has increased [6, 7]. A recent multicenter study compared the performance among radiologists, MRI findings, and radiomics-clinical models in predicting PAS disorders, highlighting the potential of radiomics approaches in this field [8]. However, radiomics is frequently limited by anisotropic resolution and low voxel statistics in current medical imaging [9]. Applying super-resolution techniques during model construction can address these limitations and improve the robustness and stability of radiomics models.

Super-resolution reconstruction (SRR) technique [10, 11]. It is a deep learning-based method for reconstructing high-resolution medical images. It can significantly enhance image resolution while maintaining existing scan times and radiation doses. Super-resolution (SR) techniques, which aim to recover higher spatial resolution in digital images, have existed since the 1980s [12]. Recently, advances in deep learning (DL) have enabled SR to demonstrate outstanding performance in medical imaging [13]. Regarding its application in MRI, some researchers [14] proposed a new convolutional neural network (CNN) model that optimizes high-frequency spatial details in short-axis cardiac MRI. In another study, SCSRN, other researchers [15] reported that a DL-SR algorithm could improve image quality for diagnosing brain morphology. Beyond its stability and reliability across spatial domains, DL-SR has gained attention for the remarkable reproducibility and robustness of radiomics features extracted in clinical applications [16, 17].

Radiomics-based approaches have been widely applied in tumor staging, early diagnosis, differentiation, prognosis prediction, and treatment evaluation across various cancers. More recently, radiomics has shown promise in obstetric imaging, including the prediction of placenta accreta spectrum disorders. Investigating whether super-resolution reconstruction can further enhance the clinical utility of radiomics for PAS prediction is therefore of considerable importance. This study aims to evaluate the value of an SRR radiomics model for diagnosing PAS. We will analyze its application in PAS diagnosis and compare its performance with conventional radiomics without SRR. Furthermore, we seek to determine whether this approach can provide new diagnostic support for PAS. This study provides clinicians with a novel diagnostic approach for PAS to improve diagnostic accuracy and enhance patient care.

In summary, while previous studies have explored radiomics for PAS diagnosis and others have applied super-resolution reconstruction (SRR) to medical imaging, the specific value of SRR-enhanced radiomics for PAS prediction remains unclear. This multicenter study aims to address this gap by:

- First, developing and validating a T2WI-based radiomics framework incorporating deep learning-based SRR (2x and 4x) for PAS prediction across three independent centers.
- Second, systematically comparing the diagnostic performance of radiomics models built on original-resolution (1x) images versus those built on 2x and 4x SRR images.
- Third, employing an automated nnUNet-based segmentation model to ensure consistency and reproducibility in defining placental regions of interest for SRR-based radiomics analysis.
- Fourth, providing evidence-based insights into whether SRR offers incremental diagnostic value for PAS within the radiomics paradigm, which has implications for future image preprocessing strategies in this clinical context.

## 2 MATERIALS AND METHODS

### 2.1 Patients

This retrospective study collected maternal MRI images and clinical data from patients with suspected PAS from three centers: First People’s Hospital of Shangqiu, Henan Province (Center A), The Second Affiliated Hospital of Shaanxi University of Chinese Medicine, Shaanxi Province (Center B), and Yan’an University Affiliated Hospital, Shaanxi Province (Center C). This retrospective study was conducted in accordance with the principles of the Declaration of Helsinki. The study protocol was approved by the Medical Ethics Committee of The Second Affiliated Hospital of Shaanxi University of Chinese Medicine, China (Approval No.: LW2023029). The need for written informed consent was waived by the ethics committee due to the retrospective nature of the study. Center A provided 480 cases, Center B provided 66 cases, and Center C provided 57 cases. The data collection period spanned from January 2016 to December 2023. Placental MRI scans were performed using a 1.5T PHILIPS 240PW system at Center A, a 3.0T Skyra (Siemens Medical Systems) at Center B, and a 1.5T GE Signa Excite HD system at Center C. The MR-T2WI scanning parameters are summarized in Table **1**.

### 2.2 Inclusion and Exclusion Criteria

#### 2.2.1 Inclusion Criteria

(1) Gestational age ≥ 28 weeks;

(2) Complete and clear MRI data meeting diagnostic criteria;

(3) Confirmation by clinical surgery or pathology.

#### 2.2.2 Exclusion Criteria

(1) Poor-quality MRI images unsuitable for diagnostic interpretation;

(2) Fetal or uterine developmental abnormalities;

(3) Coexisting pelvic pathologies (*e.g*., tumors, inflammation, vascular diseases) or other confounding conditions.

A detailed flowchart of the patient inclusion and exclusion process is provided in Fig (**1**).

It should be noted that there were no missing data for any of the clinical variables included in this analysis. As indicated in Table **2**, complete clinical records were available for all enrolled participants. *n* = 603 across the three centers. Therefore, no imputation or additional handling of missing values was required for the regression analyses or model training.

### 2.3 Automated Segmentation

An automated placental segmentation model was established using the deep-learning nnUNet framework [18], as illustrated in Fig (**2**). The model's segmentation performance was evaluated on two external validation sets, yielding Dice similarity coefficients of 0.863 and 0.883, respectively (Table **3**).

### 2.4 Radiomics

#### 2.4.1 Image Acquisition and Processing

MRI T2-weighted sagittal images were acquired from suspected PAS patients across three centers. All images underwent preprocessing and SRR to generate 2× and 4× super-resolution images. The reconstruction was performed using ONEKEY AI software with magnification factors of 2 and 4. This software employs a deep learning-based generative adversarial network (GAN) architecture specifically trained on medical images to enhance spatial resolution while preserving anatomical details. A deep transfer learning approach was applied to T2-weighted imaging (T2WI) to enhance z-axis resolution, producing preoperative 2× and 4× SR-T2WI images Fig (**3**).

#### 2.4.2 Region of Interest Segmentation

A radiologist with over ten years of experience in obstetric disease diagnosis used ITK-SNAP software (version 3.8.0, itksnap.org) to segment and outline the regions of interest (ROIs) on pre-super-resolution (S-pre) images. The ROIs were defined as the entire placental parenchyma, including the placental tissue from the basal plate to the chorionic plate, while carefully excluding adjacent structures such as the uterine wall, amniotic fluid, and fetal parts. These ROIs were then reviewed and edited by a second radiologist with 16 years of experience, who was blinded to clinical information. ROIs were manually delineated slice-by-slice along the placental boundaries on T2WI sagittal images, with care taken to avoid exceeding placental tissue boundaries. In cases where placental borders were indistinct due to artifacts or adjacent structures, consensus was reached through discussion between the two radiologists. The reproducibility of inter- and intra-observer volume of interest (VOI) segmentation was assessed using Dice similarity coefficients. Both a Dice similarity coefficient and an intraclass correlation coefficient (ICC) greater than 0.75 were considered indicative of good agreement for image segmentation and radiomic feature extraction. Post-super-resolution (S-post) images were segmented using the automated model, with results manually verified for accuracy.

It should be noted that manual segmentation was performed on pre-super-resolution (1×) images, while automated nnUNet segmentation was applied to post-super-resolution (2× and 4×) images. This methodological choice was made because manual segmentation on super-resolved images would be extremely time-consuming and prone to intra- and inter-observer variability, potentially introducing additional noise. The automated nnUNet model, once trained on manually segmented ground truth, offers consistent and reproducible segmentation across all super-resolved images. To ensure comparability between resolutions, we validated the automated segmentation performance against manual segmentation on a subset of images, achieving Dice similarity coefficients of 0.863 and 0.883 on the two external validation sets, indicating excellent agreement. Nevertheless, we acknowledge this inconsistency as a potential limitation, which we discuss further in the Limitations section.

#### 2.4.3 Feature Selection and Model Construction

Radiomic features were extracted from the ROIs, including first-order statistics (18 features), shape features (14 features), and texture features derived from the gray-level dependence matrix (GLDM, 14 features), gray-level co-occurrence matrix (GLCM, 24 features), gray-level run length matrix (GLRLM, 16 features), gray-level size zone matrix (GLSZM, 16 features), and neighboring gray tone difference matrix (NGTDM, 5 features). A total of 107 radiomic features were extracted across all categories. Feature selection was performed using Student's t-test and the Mann-Whitney U test, with a significance threshold of *p* < 0.05. The Spearman rank correlation coefficient was used to assess inter-feature correlations, and a greedy recursive elimination strategy was applied to retain features with high correlation. Least Absolute Shrinkage and Selection Operator (LASSO) regression was employed for feature selection and model construction. Ten-fold cross-validation was used to identify the optimal λ value corresponding to the minimum cross-validation error. Through this process, the number of selected features was reduced to 15, 12, and 24 for the 1x, 2x, and 4x resolution models, respectively. These selected features were linearly combined using their model coefficients as weights to generate the radiomics signature. Specifically, for each patient, the radiomics signature was calculated as the linear combination of selected feature values weighted by their respective LASSO coefficients. This signature represents a quantitative measure of the imaging phenotype associated with PAS. Subsequently, this radiomics signature was entered as the sole independent variable into a logistic regression (LR) model to construct the final risk prediction model. This approach allows for the generation of a clinically interpretable probability score (ranging from 0 to 1) representing the likelihood of PAS. The final model parameters were determined using 5-fold cross-validation to ensure stability and generalizability.

It should be noted that the initial feature screening using univariate tests (Student's t-test and Mann-Whitney U test) was performed without correction for multiple comparisons. Given the large number of extracted features, *n* = 107, this approach may increase the risk of retaining false-positive features in the initial screening step. However, we subsequently applied LASSO regression with ten-fold cross-validation for further feature selection. LASSO is a regularization technique that shrinks coefficients of less informative features to zero, thereby inherently controlling for overfitting and reducing the impact of potentially spurious associations identified in the univariate screening. This two-step strategy balances the need for comprehensive feature exploration with the mitigation of false-positive risks. Nevertheless, we acknowledge the absence of explicit multiple comparison correction as a limitation, which we discuss further in the Limitations section.

The selection of K-Nearest Neighbors (KNN), AdaBoost, and Gradient Boosting as our classification algorithms was guided by several considerations. First, these three models represent distinct families of machine learning approaches: KNN is an instance-based learner that captures local patterns in feature space; AdaBoost is an ensemble method that sequentially corrects misclassifications by weighting samples; and Gradient Boosting builds an ensemble of weak learners in a stage-wise fashion to optimize predictive performance. This diversity allows us to explore how different algorithmic paradigms perform on the radiomics feature set. Second, these models have been widely and successfully applied in previous radiomics studies for various medical imaging tasks, including PAS prediction, demonstrating their suitability for high-dimensional radiomics data. Third, compared to more complex deep learning models, these algorithms offer greater interpretability and require fewer computational resources, which aligns with our goal of developing clinically transparent and reproducible models. We acknowledge that other models, such as regularized logistic regression or more recent ensemble methods (*e.g*., XGBoost, LightGBM), could also be considered; however, the chosen set provides a representative and well-established baseline for evaluating the impact of super-resolution reconstruction on radiomics-based PAS prediction.

### 2.5 Statistical Analysis

The primary aim of the statistical analysis was to evaluate the performance of the super-resolution radiomics models for predicting PAS and to compare their diagnostic efficacy across different resolutions. Additionally, we aimed to assess the clinical characteristics associated with PAS and to validate the reproducibility of the segmentation process.

Statistical analyses were performed using SPSS (version 26.0; IBM Corp.). Quantitative data conforming to normal distribution and homogeneity of variance are presented as mean ± standard deviation (x̄ ± s) and were compared between groups using the two-sample t-test. Quantitative data not meeting assumptions of normal distribution or homogeneity of variance are presented as median (interquartile range) [M(Q)] and were compared using the Mann-Whitney U test. Categorical data were analyzed using the chi-square test. Inter-observer agreement was considered excellent when the Kappa coefficient, Dice similarity coefficient, or ICC exceeded 0.75. Binary logistic regression analysis was performed on factors showing statistically significant differences in univariate analysis, with *p* < 0.05 considered statistically significant. For model performance comparison, receiver operating characteristic (ROC) curve analysis was conducted, and the area under the curve (AUC) was calculated for each model. DeLong's test was used to compare the statistical significance of differences between AUCs of models built on 1x, 2x, and 4x super-resolution images.

## 3 RESULTS

### 3.1 Clinical Characteristics

The clinical characteristics of the training dataset are summarized in Table **2**. The collected clinical data include: age, gestational age, placenta previa, placental position, placental thickness, cervical length, history of abortion, history of cesarean section or curettage, body mass index (BMI), vaginal bleeding, and blood transfusion during surgery.

### 3.2 The Radiomics Model of 1x and 2x,4x Super-Resolution Reconstruction

**Fig. (4):**
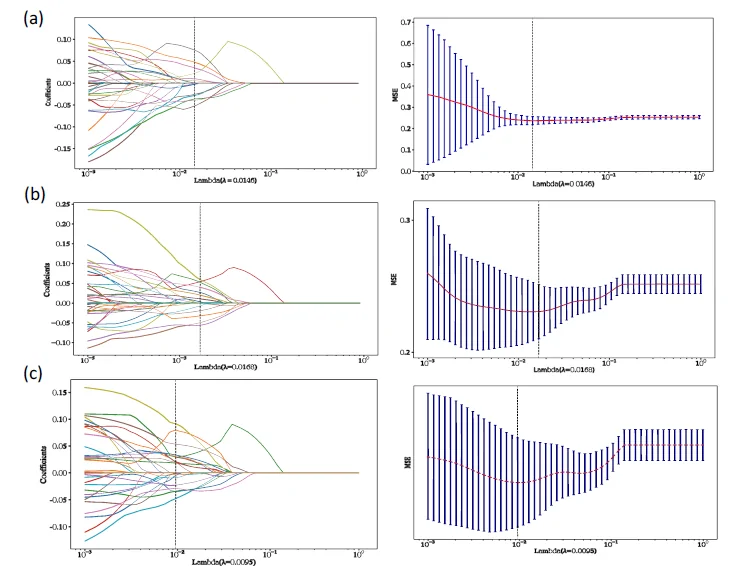
Feature selection using LASSO regression for (**a**) 1x, (**b**) 2x, and (**c**) 4x super-resolution images. For each resolution, the left column shows the cross-validation deviance (binomial deviance) as a function of the tuning parameter log(λ). The vertical dashed line indicates the optimal λ value selected by ten-fold cross-validation using the minimum deviance criterion. The right column displays the coefficient paths of the radiomics features as log(λ) varies. Each colored line represents the trajectory of a specific feature's coefficient. The vertical dashed line corresponds to the optimal λ, at which features with non-zero coefficients were retained for model construction.

The AUC values for all models in the training dataset are presented in Table **4**. Figure **4** illustrates the feature selection process in the training dataset. This process utilized LASSO regression, a method that performs both feature selection and regularization simultaneously.

### 3.3 Comparison of 1x, 2x, and 4x Super-Resolution Reconstruction

The AUC values for the KNN, AdaBoost, and Gradient Boosting models across the three centers are shown in Figs (**5**-**7**). These figures present comparisons between the training set and the test sets 1 and 2.

### 3.4 The Delong Test

DeLong's test found no statistically significant differences between 1×, 2×, and 4× SRR KNN/AdaBoost/Gradient Boosting models. (Table **5**).

## 4 DISCUSSION

This study developed an independently validated radiomics model for PAS based on SR-T2WI. The results indicate that radiomics models using 2× or 4× super-resolution reconstructed SR-T2WI did not demonstrate an advantage over models using pre-super-resolution reconstructed images. These findings suggest that while SRR represents a promising image preprocessing approach, it may not be suitable for PAS, and further research is needed to identify more effective image preprocessing methods for PAS prediction.

Currently, ultrasound remains the preferred modality for diagnosing PAS disorders [19-21], while MRI is recommended as an effective supplementary examination [22, 23]. However, diagnostic accuracy remains unsatisfactory, necessitating alternative methods to improve preoperative evaluation. Radiomics, with its high-throughput data mining capability, provides extensive additional information for clinical application [24]. In previous studies, researchers [25, 26] have applied SRR to medical images and compared radiomic features before and after reconstruction. Many studies [27, 28] have reported that SRR significantly improves diagnostic efficacy. The primary distinction between original and super-resolution reconstructed images lies in enhanced image resolution a difference not readily discernible to the naked eye. Consequently, numerous investigations [10, 29] have employed artificial intelligence methods, particularly radiomics, for analysis. T2WI-based radiomics models have demonstrated consistently strong performance [30, 31]. However, due to the physical principles of T2WI, in-plane resolution exceeds through-plane resolution [32]. Consequently, the spatial resolution along the z-axis (z-resolution) is anisotropic, which may adversely affect 3D radiomics characterization. This limitation can be mitigated through SR techniques [33]. Among various SR methods proposed for medical imaging, deep learning-based SR (DL-SR) is preferred due to its ability to overcome high-frequency information loss and edge blurring while enhancing resolution [34]. Several recent studies have established a foundation for applying DL-SR to radiomics analysis [17, 35, 36]. Unlike these preliminary investigations, which primarily established technical feasibility, this study specifically evaluated whether super-resolution reconstructed radiomics improves the diagnostic efficacy for PAS. We performed 2× and 4× SRR on acquired T2WI sagittal images and compared radiomics models derived from pre- and post-reconstruction images. Although previous research suggests that super-resolution reconstructed radiomics can enhance diagnostic performance, the present study found that while SR techniques significantly improved image resolution, SR-T2WI radiomics did not significantly improve diagnostic efficacy for PAS.

In this study, we evaluated the performance of super-resolution reconstructed radiomics models for diagnosing PAS. In the training dataset, the AUC for the pre-super-resolution radiomics (1×) model was 0.794, while the AUC values for the 2× and 4× post-super-resolution radiomics models were 0.794 and 0.863, respectively. DeLong's test demonstrated that the post-super-resolution radiomics models (2×/4×) did not outperform the pre-super-resolution model (1×). Although SRR can produce clearer images, its application did not significantly improve the diagnostic accuracy for PAS. This may be because PAS diagnosis depends heavily on physician experience and image interpretation, and SRR may not provide substantial additional diagnostic information. Notably, the AUC values on the external validation sets (Center B/C) were lower than those in the training set following SRR. We identified several potential reasons for this discrepancy, including: (1) differences in sample distribution between the training and validation sets, and (2) the limited sample size in the external validation sets.

While calibration and decision-curve analyses were not performed in this study, the clinical implications of our models can still be inferred from the reported performance metrics. For example, at the optimal threshold determined by Youden's index, the 1x model achieved a sensitivity of 85.7% and specificity of 82.3% in the training cohort, suggesting that it could potentially aid in identifying high-risk patients who may benefit from specialized prenatal counseling and delivery planning. However, we acknowledge that prospective validation with calibration assessment and DCA would be necessary before clinical implementation.

## 5 STUDY LIMITATIONS

This study has several limitations. First, regarding feature selection and model construction, our initial univariate screening was performed without correction for multiple comparisons, which may increase the risk of retaining false-positive features despite subsequent LASSO regularization. Additionally, while we selected KNN, AdaBoost, and Gradient Boosting to represent diverse algorithmic families, we did not include other potentially effective models such as regularized logistic regression or modern ensemble methods (*e.g*., XGBoost, LightGBM) that may offer advantages in calibration or efficiency. Second, several factors may limit the statistical power and generalizability of our findings. The external validation cohorts (Center B: n=66; Center C: n=57) were relatively small, potentially reducing the power to detect modest but clinically meaningful differences between resolutions. Although DeLong tests showed no significant improvements with super-resolution, we cannot entirely rule out the possibility that clinically relevant differences exist but were not detected due to insufficient power. Furthermore, while our multicenter design enhances generalizability, we did not perform formal inter-scanner or inter-sequence feature robustness analyses. The moderate performance in external cohorts may partly reflect feature variability across different scanners and protocols, highlighting the need for harmonization techniques such as ComBat in future studies. Third, methodological inconsistencies may introduce bias. Manual segmentation was used for pre-SR images, while automated nnUNet segmentation was applied to post-SR images. Although we validated automated against manual segmentation with high Dice coefficients (0.863-0.883), this inconsistency remains a potential source of bias when comparing across resolutions. Moreover, the automated segmentation model's generalization has only been preliminarily validated on two external datasets and requires broader testing. Fourth, our evaluation focused primarily on discriminative performance (AUC) without quantitative comparison against standard MRI assessment by radiologists or existing clinical-radiological models. We acknowledge that the absence of calibration metrics and decision-curve analysis limits the assessment of clinical utility in this study. Future prospective studies with larger, more diverse cohorts are warranted to perform these analyses and validate the models' clinical applicability. The absence of these analyses limits our ability to demonstrate the true clinical added value of our radiomics models. Future studies should address these limitations by incorporating more stringent feature selection methods, expanding classifier comparisons, including larger and more diverse external cohorts, applying harmonization techniques for multi-scanner data, ensuring consistent segmentation methods, and integrating calibration metrics and decision-curve analysis alongside direct comparisons with clinical practice.

### 5.1 Balancing Model Performance and Explainability: The Role of XAI

While deep learning (DL) architectures often achieve superior predictive performance compared to shallow models, their “black-box” nature limits inherent explainability a critical requirement in clinical applications for building trust and ensuring patient safety. Explainable Artificial Intelligence (XAI) addresses this gap by providing insights into model decision-making without substantially compromising accuracy [37]. Techniques such as SHAP, LIME, and Grad-CAM have been increasingly applied in medical imaging to highlight influential features driving predictions [38]. In the context of PAS prediction, although our study focused on interpretable shallow models, future research could integrate XAI with more complex DL architectures. This approach would harness potential performance gains while maintaining the transparency necessary for clinical adoption, representing a promising direction for advancing the field.

## CONCLUSION

In conclusion, although SRR techniques can significantly enhance image resolution, this study found that their application did not significantly improve the diagnostic efficacy for PAS. Therefore, while super-resolution remains a valuable preprocessing and technical enhancement tool in other domains, it does not appear to be applicable for PAS diagnosis based on current evidence. For future PAS research, this study suggests that performing SRR on images may not be necessary, and efforts should instead focus on exploring more effective diagnostic approaches.

## Figures and Tables

**Fig (1):**
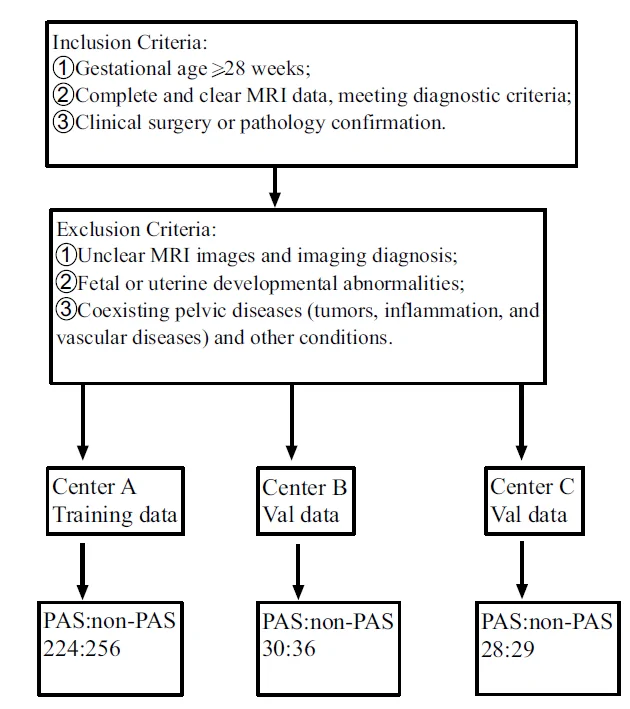
The flow chart for the inclusion and exclusion criteria of patients.

**Fig. (2):**
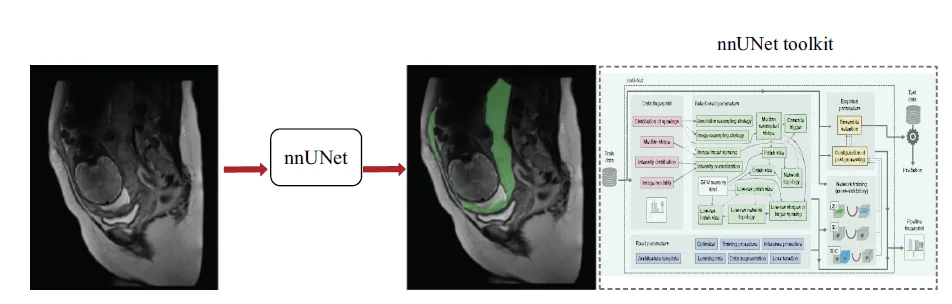
Placenta segmentation process using nnUNet. Left: Original T2WI sagittal image with manual ROI delineation (green contour). Right: Schematic diagram of the nnUNet toolkit architecture, illustrating its self-configuring pipeline that automatically adapts network architecture, preprocessing, and training strategies based on dataset properties. The segmented example shown is a PAS case, with the automated segmentation result (green contour) overlaid on the original image.

**Fig. (3):**
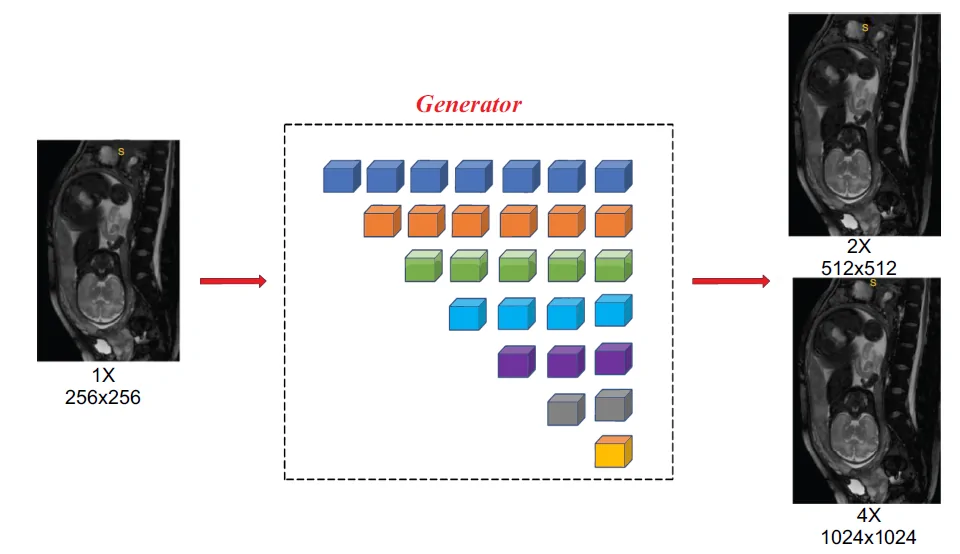
Schematic workflow of super-resolution reconstruction (SRR) using ONEKEY AI software. The central “Generator” block represents the ONEKEY AI software, which utilizes a GAN-based deep learning model trained on medical images to enhance resolution. Input images (1x) are processed through the generator to produce 2x and 4x super-resolution images. The right panel shows representative sagittal T2WI slices at 2x and 4x resolutions.

**Fig. (5):**
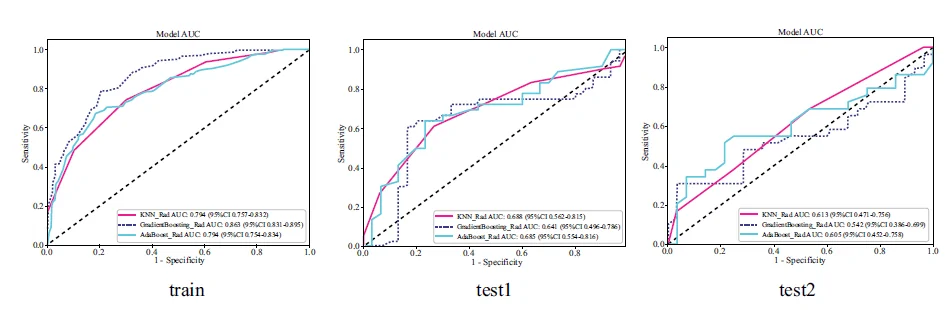
The 1× AUC of the comparison of KNN, AdaBoost, and Gradient Boosting models in the three centers.

**Fig. (6):**
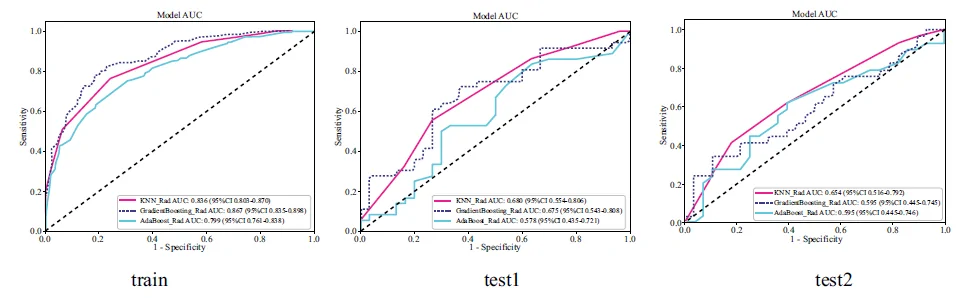
The 2× AUC of the comparison of KNN, AdaBoost, and Gradient Boosting models in the three centers.

**Fig. (7):**
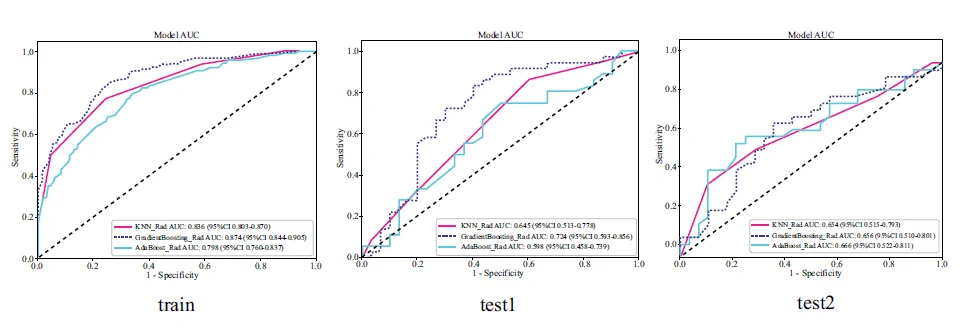
The 4× AUC of the comparison of KNN, AdaBoost, and Gradient Boosting models in the three centers.

**Table 1:** MR-T2WI scanning parameters.

**Parameters**	**Center A** **(Philips Balance)**	**Center B** **(Siemens Haste)**	**Center C** **(GE Fiesta)**
FOV	400×400mm	420×320mm	400×360mm
Interval	1mm	1mm	1mm
Thickness	5mm	5mm	5mm
Matrix	248×211	320×75%	256×256
TR	4.0ms	1400ms	3.7ms
TE	2.0ms	95ms	1.6ms
NEX	1	1	2
Times	14s	44s	16s

**Table 2:** Clinical characteristics of pas and non-pas in training datasets (Center A).

**Parameters**	**(-)** **n=224**	**(+)** **n=256**	***t*/*χ^2^***	***p***
**Age**	31.52±4.056	31.26±4.327	0.678	0.498
Gestational age	37.390±2.1669	36.384±2.5322	4.643	<0.001
Gestational age1				
0	2	6	1.535	0.215
1	222	250		
**Placenta previa**			93.828	<0.001
No	134	47		
Partial	19	26		
Complete	65	178		
Low	6	5		
**Placental position**			31.891	<0.001
Anterior-Lateral	13	25		
Anterior-Posterior-Lateral	6	24		
Anterior	110	76		
Lateral	8	5		
Anterior-Posterior	18	41		
Posterior-Lateral	11	21		
Posterior	58	64		
Placental thickness (cm)	4.524±1.0165	4.591±1.1147	-0.679	0.478
Cervical length (cm)	2.764±0.6406	2.823±0.7161	-0.938	0.349
History of abortion			4.274	0.640
0	76	66		
1	67	89		
2	45	60		
3	26	31		
4	7	7		
5	2	2		
6	1	1		
History of cesarean section or curettage			34.343	<0.001
0	49	60		
1	41	101		
2	112	85		
3	21	8		
4	1	2		
BMI	28.651±3.5307	28.076±3.4796	1.796	0.073
Vaginal bleeding			37.350	<0.001
No	168	122		
Yes	56	134		
Blood transfusion during surgery			60.589	<0.001
No	187	127		
Yes	37	129		

**Note:** Data were complete for all variables presented in this table for the included participants. Gestational age1 represents binary gestational age grouping stratified by clinical management criteria for placenta accreta spectrum disorders. Category 0 indicates 28–34 weeks of gestation, and category 1 indicates gestational age ≥35 weeks. Continuous gestational age data are presented as mean±standard deviation for quantitative comparison, while this binary variable is added as a supplementary categorical analysis to explore the distribution of preterm risk between PAS and non-PAS cohorts.

**Table 3:** Segmentation performance (dice and related metrics) on the two external validation sets.

**Method**	**Test Set 1 (n=66)**				**Test Set 2 (n=57)**			
	Dice	Jaccard	Recall	Precision	Dice	Jaccard	Recall	Precision
nnU-Net	0.863	0.772	0.864	0.874	0.883	0.795	0.885	0.889

**Note:** Test Set 1 corresponds to Center B; Test Set 2 corresponds to Center C.

**Table 4:** AUC values for all models in the training (Center A) and external validation (Center B and C) datasets.

**Model name**	-	**Accuracy**	**AUC(95%CI)**	**Sensitivity**	**Specificity**	**PPV**	**NPV**	**F1**	**Threshold**	-
KNN	Train	0.677	0.794(0.7565-0.8323)	0.484	0.897	0.844	0.604	0.615	0.600	1X
	Test 1	0.576	0.688(0.5623-0.8146)	0.278	0.933	0.833	0.519	0.417	0.600	
	Test 2	0.561	0.613(0.4709-0.7557)	0.345	0.786	0.625	0.537	0.444	0.600	
AdaBoost	Train	0.733	0.794(0.7542-0.8336)	0.656	0.821	0.808	0.676	0.724	0.510	
	Test 1	0.682	0.685(0.5536-0.8158)	0.611	0.767	0.759	0.622	0.677	0.506	
	Test 2	0.632	0.605(0.4525-0.7581)	0.483	0.786	0.700	0.595	0.571	0.514	
Gradient Boosting	Train	0.790	0.863(0.8305-0.8947)	0.785	0.795	0.814	0.764	0.799	0.513	
	Test 1	0.697	0.641(0.4956-0.7859)	0.583	0.833	0.808	0.625	0.677	0.571	
	Test 2	0.614	0.542(0.3863-0.6986)	0.276	0.964	0.889	0.562	0.421	0.650	
KNN	Train	0.706	0.836(0.8026-0.8797)	0.504	0.937	0.902	0.623	0.647	0.600	2X
	Test 1	0.561	0.680(0.5541-0.8061)	0.333	0.833	0.706	0.510	0.453	0.600	
	Test 2	0.544	0.654(0.5157-0.7922)	0.172	0.929	0.714	0.520	0.278	0.800	
AdaBoost	Train	0.715	0.799(0.7606-0.8381)	0.629	0.812	0.793	0.657	0.702	0.506	
	Test 1	0.606	0.578(0.4354-0.7211)	0.722	0.467	0.619	0.583	0.667	0.493	
	Test 2	0.596	0.595(0.4446-0.7463)	0.586	0.607	0.607	0.586	0.596	0.494	
Gradient Boosting	Train	0.794	0.867(0.8350-0.8980)	0.777	0.812	0.826	0.762	0.801	0.526	
	Test 1	0.667	0.675(0.5433-0.8076)	0.694	0.633	0.694	0.633	0.694	0.511	
	Test 2	0.596	0.595(0.4451-0.7446)	0.310	0.893	0.750	0.556	0.439	0.625	
KNN	Train	0.710	0.836(0.8026-0.8700)	0.500	0.951	0.921	0.625	0.648	0.600	4X
	Test 1	0.591	0.645(0.5126-0.7782)	0.556	0.633	0.645	0.543	0.597	0.400	
	Test 2	0.526	0.654(0.5145-0.7933)	0.103	0.964	0.750	0.509	0.182	0.800	
AdaBoost	Train	0.731	0.798(0.7597-0.8373)	0.789	0.665	0.729	0.734	0.758	0.500	
	Test 1	0.621	0.598(0.4577-0.7366)	0.667	0.567	0.649	0.586	0.658	0.493	
	Test 2	0.649	0.666(0.5216-0.8110)	0.517	0.786	0.714	0.611	0.600	0.501	
Gradient Boosting	Train	0.796	0.874(0.8441-0.9047)	0.824	0.763	0.799	0.792	0.812	0.500	
	Test 1	0.712	0.724(0.5926-0.8556)	0.806	0.600	0.707	0.720	0.753	0.488	
	Test 2	0.649	0.656(0.5104-0.8011)	0.655	0.643	0.655	0.655	0.655	0.562	

**Table 5:** DeLong's test for comparison of AUC among models with 1×, 2×, and 4× super-resolution.

**Model name**	**Train**		**Test 1**		**Test 2**	
	***Z***	***P***	***Z***	***P***	***Z***	***P***
1x KNN *vs* 2x KNN	1.133	0.2573	0.413	0.6796	0.381	0.7030
1x KNN *vs* 4x KNN	1.125	0.2607	0.518	0.6047	0.114	0.9096
2x KNN *vs* 4x KNN	0.101	0.9198	1.123	0.2613	0.626	0.5311
1x AdaBoost *vs* 2x AdaBoost	0.368	0.7127	0.820	0.4124	0.332	0.7398
1x AdaBoost *vs* 4x AdaBoost	0.0595	0.9526	1.442	0.1494	0.840	0.4011
2x AdaBoost *vs* 4x AdaBoost	0.309	0.7569	0.481	0.6302	0.364	0.7157
1xGradient Boosting *vs* 2xGradient Boosting	0.766	0.4436	1.145	0.2524	0.964	0.3348
1xGradient Boosting *vs* 4xGradient Boosting	0.444	0.6572	0.330	0.7412	2.144	0.0321
2xGradient Boosting *vs* 4xGradient Boosting	0.265	0.7913	1.332	0.1829	1.503	0.1329

## Data Availability

The data and supportive information are available within the article.

## LIST OF ABBREVIATIONS

AUC: Area under the curve
BMI: Body mass index
CI: Confidence interval
CNN: Convolutional neural network
DL: Deep learning
FOV: Field of view
GAN: Generative adversarial network
GLCM: Gray-level co-occurrence matrix
GLDM: Gray-level dependence matrix
GLRLM: Gray-level run length matrix
GLSZM: Gray-level size zone matrix
Grad-CAM: Gradient-weighted class activation mapping
ICC: Intraclass correlation coefficient
KNN: K-nearest neighbors
LASSO: Least absolute shrinkage and selection operator
LIME: Local interpretable model-agnostic explanations
NEX: Number of excitations
NGTDM: Neighboring gray tone difference matrix
PAS: Placenta accreta spectrum
ROC: Receiver operating characteristic
ROI: Region of interest
SHAP: SHapley Additive exPlanations
SR: Super-resolution
SRR: Super-resolution reconstruction
VOI: Volume of interest
XAI: Explainable artificial intelligence

## STANDARDS OF REPORTING

STROBE guidelines were followed.
